# Carbon nanoonion-ferrocene conjugates as acceptors in organic photovoltaic devices†

**DOI:** 10.1039/c9na00135b

**Published:** 2019-07-03

**Authors:** Diana M. Bobrowska, Halyna Zubyk, Elzbieta Regulska, Elkin Romero, Luis Echegoyen, Marta E. Plonska-Brzezinska

**Affiliations:** Institute of Chemistry, University of Bialystok Ciolkowskiego 1K 15-245 Bialystok Poland; University of Texas at El Paso 500 W University Ave., Chemistry and Computer Science Bldg. #2.0304 El Paso TX 79968-8807 USA; Department of Organic Chemistry, Faculty of Pharmacy with the Division of Laboratory Medicine, Medical University of Bialystok Mickiewicza 2A 15-222 Bialystok Poland marta.plonska-brzezinska@umb.edu.pl +48 85 748 5683

## Abstract

Many macromolecular systems, including carbon nanostructures (CNs), have been synthesized and investigated as acceptors in photovoltaic devices. Some CNs have shown interesting electrochemical, photophysical and electrocatalytic properties and have been used in energy and sustainability applications. This study focuses on the covalent functionalization of carbon nanoonion (CNO) surfaces with ferrocene moieties to obtain donor–acceptor systems involving CNOs as acceptors. The systems were synthesized and characterized by infrared, Raman, UV-vis and fluorescence spectroscopies, thermogravimetric analysis, scanning electron microscopy, nitrogen adsorption and electrochemical measurements. The HOMO–LUMO levels were calculated to evaluate the possibility of using these systems in photoactive devices. In this study, for the first time, the CNO-based derivatives were applied as acceptors in the active layer of photovoltaic devices. This study is the first to use large CNO-based derivatives as acceptors in organic photovoltaic devices, and a power conversion efficiency as high as 1.89% was achieved.

## Abstract 2

This study is the first to use carbon nanoonion-based derivatives as acceptors in organic photovoltaic devices (PCE = 1.89%).

## Graphical abstract

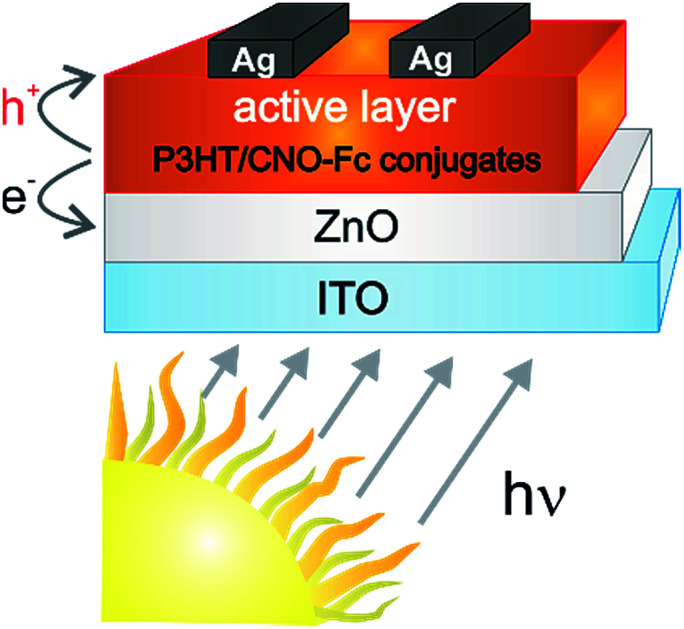

## Introduction

The rational design of molecular-based materials for catalysis, electronic and photonic applications and for the preparation and integration of multifunctional molecules into supramolecular architectures is currently a topic of great interest. In recent years, carbon nanostructured-based donor–acceptor dyads have been widely explored for organic photovoltaic (OPV) applications. OPV devices are mainly composed of an active layer, a charge-selective layer and a charge-conductive layer. The active layer, which is composed of a polymer matrix and carbon nanostructures (CNs), allows for exciton dissociation in a strong electric field with CNs as the electron acceptor/transporter.^1^ Frequently used donor moieties include polythiophenes,^2,3^ phthalocyanines,^4,5^ porphyrins^6,7^ and ferrocenes.^8–10^

Since 1994, C_60_ and its derivatives have been among the most frequently used acceptors in heterojunction OPV cells.^11–14^ Upon electron reduction, C_60_ presents a low reorganization energy.^15^ This property is due to the spherical rigid carbon framework and the delocalized π-electron system that contribute to the stabilization of the incoming charge with minimal structural or solution polarization changes.^16^ The delocalization of electrons or holes within the spherical carbon framework (diameter = 7.5 Å) offers unique opportunities for the stabilization of charges.^17^ Therefore, C_60_ can lead to fast photoinduced charge-separated (CS) states. Many reports have described the application of larger nanostructures, such as carbon nanotubes (CNTs)^18–21^ and endohedral fullerenes, instead of fullerenes.^22–24^ A promising direction proposed to improve cell efficiency is the incorporation of nanostructures, such as CNTs, which may facilitate charge separation and transport to the electrodes.^25^

Multi-shelled fullerenes, also known as carbon nanoonions (CNOs), can also be used as the acceptor in photovoltaic systems. Because of their unique spherical structures, CNOs can readily be covalently and non-covalently integrated into macromolecular systems due to their sp^2^-hybridized carbons.^26^ CNOs prepared from nanodiamond particles (NDs, 5 nm diameter) *via* thermal annealing process consist of 6 to 8 layers and are 5–6 nm in diameter.^27,28^ These spherical CNs possess very interesting physico-chemical properties, including electronic ones. The spherical CNOs obtained from NDs are paramagnetic and have unpaired electrons on their surfaces.^29^ The conductance of thiol-functionalized CNOs (synthesized at 1650 °C) in a molecular junction were investigated using scanning tunnelling microscopy.^30^ The study demonstrated that the electron transmission through CNOs occurred by a tunnelling mechanism, and the values were comparable to those of metallic nanowires. These unusual properties of CNOs prompted us to use them in OPVs as acceptor moieties in the active layer.

Some attempts have already been made to apply CNOs in photoactive devices. Since then, to the best of our knowledge, no reports on the use of CNOs as acceptor in the active layer of OPVs have been demonstrated.^31,32^ Notably, these CNs were used as hole collection layers in zinc-phthalocyanine-based OPV solar cells. The photocurrent was observed to increase by a factor of 5.5 over that of solar devices without CNOs.^31^ The highest power conversion efficiency (PCE) was up to 6.9 × 10^−2^%.^31^ Analogously, a device based on a crystalline perovskite film with oxidized CNOs incorporated into the hole transporting layer together with poly(3,4-ethylenedioxythiophene):polystyrene sulphonate (PEDOT:PSS), resulted in a significant enhancement of the PCE from 11.07 to 15.26%.^32^ Additionally, larger CNO structures (30 nm in diameter) were used in dye-sensitized solar cells as a counter electrode, and the PCE was comparable to that obtained with the commonly used platinum one.^33^

In the present study, we report the synthesis and photo- and electrochemical behaviour of some CNO-based systems prepared using different strategies. Five derivatives containing CNOs and covalently linked ferrocene derivatives were obtained, and their compositions were determined by several experimental methods. For the first time, the potential of such systems as acceptors in active layers in OPV devices is reported.

## Results and discussion

### Synthesis and spectroscopic characterization of CNO-Fc derivatives

The reactivity of CNOs lies between that of C_60_ and graphite. C_60_ is relatively reactive, while graphite is extremely inert towards chemical functionalization. The reactivity of CNOs depends on the number of graphitic layers, since increasing the number of layers leads to a decrease in curvature, reactivity and solubility.^29^ In this report, we used spherical CNOs with diameters of 5 to 6 nm obtained by the thermal annealing of NDs.

**Scheme 1:**
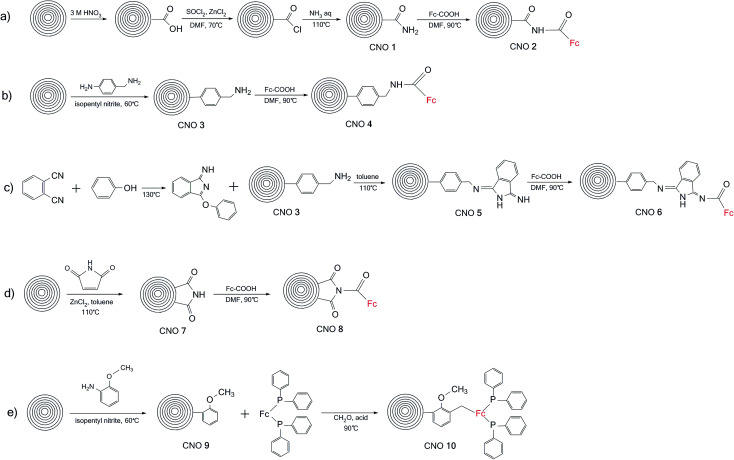
Syntheses of the CNO-Fc derivatives: (a) CNO 1 and CNO 2, (b) CNO 3 and CNO 4, (c) CNO 5 and CNO 6, (d) CNO 7 and CNO 8, and (e) CNO 9 and CNO 10.

The synthetic procedures used to prepare the carbon nanoonion-ferrocene (CNO-Fc) systems are shown in Scheme 1. These derivatives were synthesized using five different approaches. The details of the synthetic procedures are described in the Experimental section. Briefly, in the first approach, CNOs were functionalized with ferrocenecarboxylic acid (CNO 2) *via* an acylation reaction of the amino-CNO derivative (CNO 1) (Scheme 1a). CNOs with carboxamide groups were obtained using oxidized-CNOs (ox-CNOs), which were functionalized according to a procedure previously described.^26,34^ In the second approach, CNO 4 was prepared in two steps first involving the functionalization of pristine CNOs with 4-aminobenzylamine (CNO 3) and then a reaction with ferrocenecarboxylic acid, as shown in Scheme 1b. The preparation of CNO 5 was performed using the reaction of 1,2-dicyanobenzene with phenol without any solvent, followed by reaction with CNO 3 in toluene. The CNO 5 derivative was further reacted with ferrocenecarboxylic acid. CNO 7 was obtained by the Diels–Alder addition of maleimide to pristine CNOs. Ferrocenecarboxylic acid was subsequently added to CNO 7, to obtain CNO 8 (Scheme 1d). The combination of 1,1′-bis(diphenylphosphino)-ferrocene with CNO 9 (Scheme 1e) resulted in the formation of CNO 10. The reaction was performed in the presence of isopentyl nitrite as a catalyst.

**Fig. 1:**
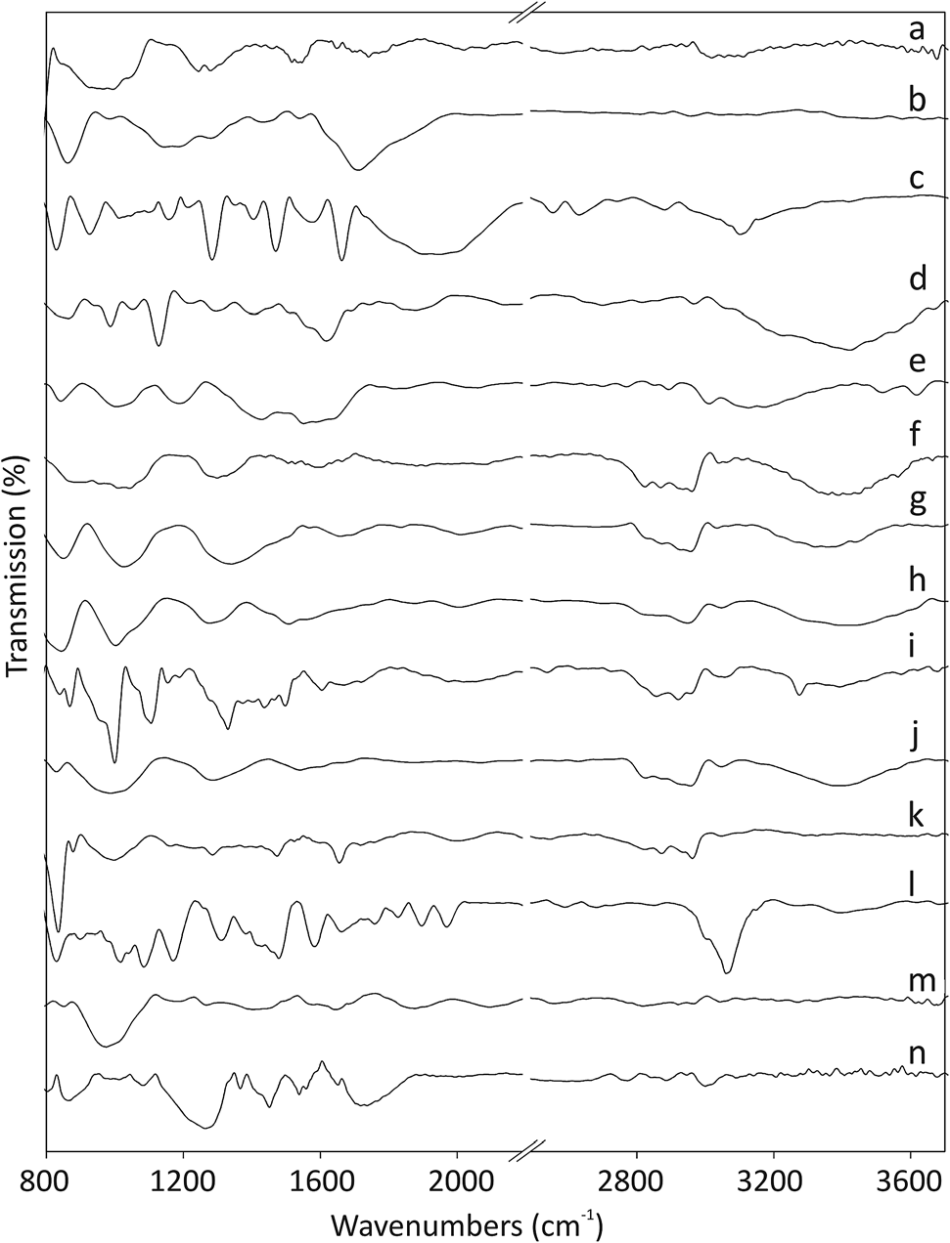
FT-IR spectra of (a) CNOs, (b) ox-CNOs, (c) ferrocenecarboxylic acid, (d) CNO 1, (e) CNO 2, (f) CNO 3, (g) CNO 4, (h) CNO 5, (i) CNO 6, (j) CNO 7, (k) CNO 8, (l) 1,1′-bis(diphenylphosphino)-ferrocene, (m) CNO 9, and (n) CNO 10.

The Fourier-transform infrared (FT-IR) spectra of pristine and functionalized CNOs are shown in Fig. 1. The ferrocene compounds (ferrocenecarboxylic acid and 1,1′-bis(diphenylphosphino)-ferrocene) were used as controls for comparison (see spectra Fig. 1c and 1l, respectively). The band at 1545 cm^−1^ for CNOs (Fig. 1a) corresponds to the C=C stretching vibration. Two bands appearing at approximately 3020 and 995 cm^−1^ arise from =C–H and =CH_2_ stretching vibrations, which indicate the presence of defects in the CNO structures.^29^ Additionally, the two bands appearing at 1741 and 1245 cm^−1^ are assigned to C=O bonds, which likely form during the annealing of CNOs in air at 400 °C.^35^ The main peaks in the IR spectrum of ox-CNO appear at 1710 and 1280 cm^−1^ and belong to the stretching vibrations of the C–O bonds formed during the oxidation process of graphite-like layers in the CNO structure (Fig. 1b).^29^

The intensities of the peaks in the 1440–1540 cm^−1^ region, associated with the C=C stretching vibrations of the rings, decrease upon oxidation of the C=C bonds. Ferrocene derivatives exhibit peaks in the 820–1960 cm^−1^ region,^36,37^ and the pattern depends on the molecular structure of the complex. The intense peaks at 1470, 1580 and 1950 cm^−1^ are assigned to the C=C and C–H stretching vibrations of the rings. The band appearing at 1158 cm^−1^ and the two peaks at 926 and 830 cm^−1^ are assigned to C–H in-plane bending and out-of-plane deformations, respectively. The appearances of a broad band at approximately 3100 cm^−1^ for the O–H stretching vibration and strong peaks at approximately 1680 and 1285 cm^−1^ arising from C=O stretching vibrations indicate the presence of carboxylic acid groups on the carbon nanostructures (Fig. 1c). After the first stage of the reaction, the CNO derivatives (CNO 1, CNO 3, CNO 5, and CNO 7) contain NH and NH_2_ groups (reactions a, b, c, and d; Scheme 1). The presence of peaks in the range of 3390–3420 cm^−1^, which correspond to N–H stretching vibration, can be used to monitor the presence of NH and NH_2_ groups. For these compounds, other peaks characteristic of the pristine CNOs are also observed. In the case of Fc-CNO derivatives CNO 2, CNO 4, and CNO 6, the stretching vibrations of N–H groups are noted. The bands appearing in the range from 1020–1295 cm^−1^ arise from C–N stretching vibrations, and they are discerned for all products from CNO 1 to CNO 8. The vibrational bands observed at frequencies in the range from 1665–1800 cm^−1^ for C=O bonds indicate the successful modification of CNOs by ferrocenecarboxylic acid groups.

The reaction with the 1,1′-bis(diphenylphosphino)-ferrocene derivative results in changes in the FT-IR spectrum (Fig. 1l). The bands appearing at approximately 3060 and 830 cm^−1^ and in the range from 1480 to 1660 cm^−1^ are assigned to C–H and C=C stretching vibrations of benzene rings, and those at 1970 and 1895 cm^−1^ are also assigned as the C=C stretching vibrations of the benzene rings. The bands near 1085 cm^−1^ arise from C–P stretching vibrations. The spectrum of CNO 9 is shown in Fig. 1m and contains bands at 2820 and 2920 (CH_3_ bending vibrations), 2095 and 1875 (C=C stretching vibrations of alkenes), 1655 and 1405 (C=C stretching vibrations of the benzene rings), 1270 (C–O stretching vibrations) and 975 (=C–H out-of-plane deformation vibrations) cm^−1^. The characteristic peaks at approximately 3000 and 865 cm^−1^, and at 1560 and 1450 cm^−1^ observed for CNO 10 correspond to the stretching and bending of C–H and the stretching bonds of C=C groups in benzene rings (Fig. 1n). Therefore, the peaks at 1720 and 1265 cm^−1^ represent the stretching of C–O groups, and the peaks at approximately 1080 cm^−1^ are assigned to C–P stretching vibrations.

**Fig. 2:**
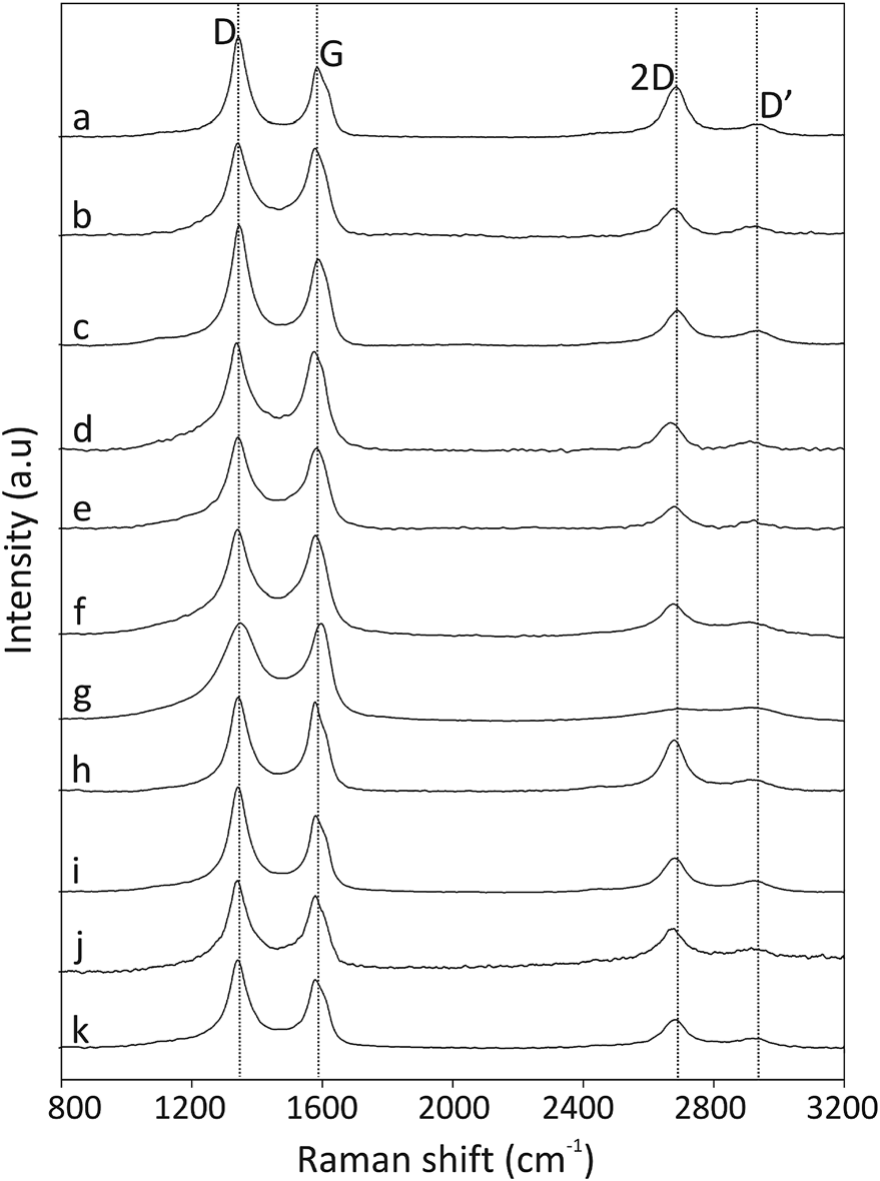
Raman spectra (recorded at *λ*_ext_ = 514 nm) of (a) CNOs, (b) CNO 1, (c) CNO 2, (d) CNO 3, (e) CNO 4, (f) CNO 5, (g) CNO 6, (h) CNO 7, (i) CNO 8, (j) CNO 9, and (k) CNO 10.

The Raman spectra excited at 514.5 nm of pristine and functionalized CNOs are shown in Fig. 2, and the data are collected in Table 1. For CNOs, the strongest features are observed at 1337 (D line) and 1576 cm^−1^ (G line), and the shifts of these bands for functionalized CNOs are summarized in Table 1. To measure the change in the position and intensity of Raman peaks, the spectra were normalized to the intensity of the G band and fitted by four Lorentz-shape components.^38^ For all pristine and functionalized CNOs, the D and G lines are intense and broad, and the D band is stronger than the G band. The functionalization of the CNs affects the G and D bands' positions and intensities. Decreasing the number of aromatic rings in the structure leads to a reduction in the frequency and intensity of the D band (Table 1). An increase in the line width of the D band also suggests an increase in the disorder of the functionalized CNOs. The G band appears at 1576 cm^−1^ for CNOs (Fig. 2a) and shifts to higher frequencies for the functionalized CNOs (Fig. 2b–k; see also Table 1). These observations can be explained by a decrease in the size of the aromatic segments upon functionalization.^39^ The intensity ratios (*I*_D_/*I*_G_) for pristine CNOs and their derivatives were also calculated (Table 1).

**Table 1:** Best-fit frequencies for D and G bands obtained at a 514 nm laser excitation energy and the relative *I*_D_/*I*_G_

Sample	D (cm^−1^)	G (cm^−1^)	2D (cm^−1^)	*I*_D_/*I*_G_
CNO	1337	1576	2678	1.40
CNO 1	1341	1578	2677	1.17
CNO 2	1345	1589	2688	1.41
CNO 3	1337	1576	2667	1.23
CNO 4	1341	1581	2676	1.35
CNO 5	1341	1579	2676	1.16
CNO 6	1349	1596	2692	1.40
CNO 7	1342	1578	2680	1.18
CNO 8	1342	1579	2680	1.61
CNO 9	1340	1578	2675	1.34
CNO 10	1342	1579	2684	1.53

The intensity ratio *I*_D_/*I*_G_ is observed to decrease after the first step of the reactions (Scheme 1) for CNO 1, CNO 3, CNO 5, CNO 7 and CNO 9. These observations indicate a decrease in the number of sp^2^-hybridized atoms after the covalent functionalization of CNOs. An increase in the intensity of *I*_D_/*I*_G_ for CNO 2, CNO 4, CNO 6, CNO 8, and CNO 10 results from an increase in sp^2^-hybridized C=C bonds in the structure due to the addition of ferrocene to the CNO surface. All samples also exhibit broad 2D bands at approximately 2680 cm^−1^. The 2D band is an overtone of the D band and is sensitive to changes in the electronic structure of the molecules.^40,41^

### Thermogravimetric analysis of CNO-Fc derivatives

**Fig. 3:**
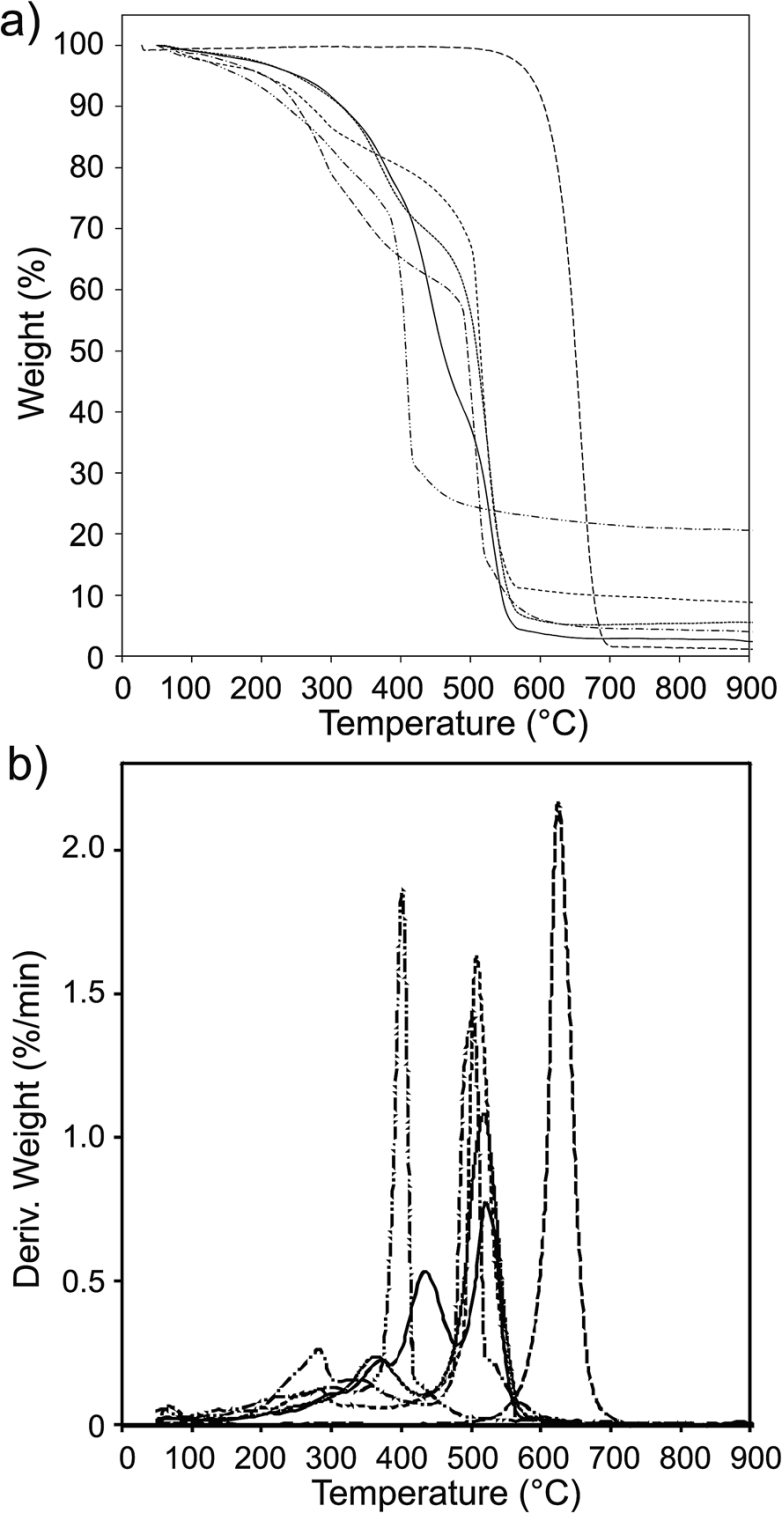
(a) TGA and (b) DTG curves of pristine CNOs (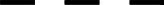), CNO 2 (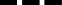), CNO 4 (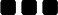), CNO 6 (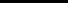), CNO 8 (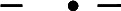) and CNO 10 (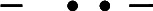) under an air atmosphere at 15 °C min^−1^.

The thermal stability of the pristine CNOs and the ferrocene-derivatives was probed using thermogravimetric analysis (TGA-DTG), and the results are presented in Fig. 3 and are summarized in Table 2. The pristine CNOs are thermally stable up to 500 °C under an air atmosphere, with inflection and end temperatures at 633 and 700 °C, respectively. The thermal stabilities of the CNO-Fc derivatives are reduced by approximately 150 °C compared with that of the unmodified CNOs (Fig. 3 and Table 2).

**Table 2:** Thermogravimetric analysis of pristine CNOs and CNO-Fc derivatives

Sample	Peak	Onset *T* (°C)	Inflection *T* (°C)	End *T* (°C)	Weight loss (%)
CNO	1	500	633	700	98
CNO 2	1	176	259	600	89
	2		506		
CNO 4	1	100	365	633	91
	2		524		
CNO 6	1	112	435	630	94
	2		524		
CNO 8	1	100	347	630	92
	2		500		
CNO 10	1	100	300	565	78
	2		400		

**Fig. 4:**
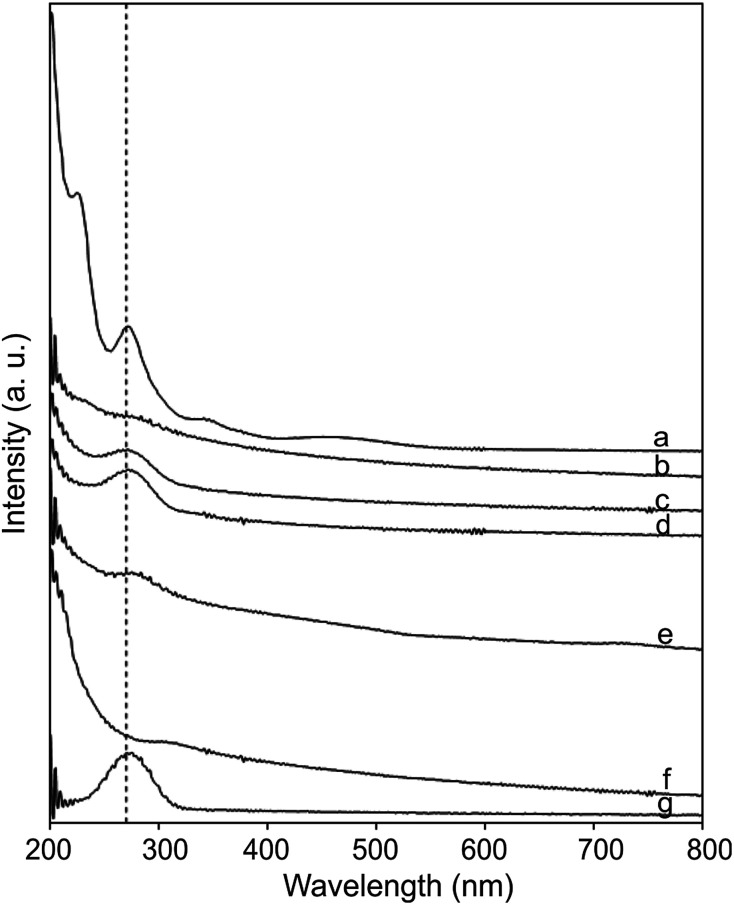
UV-vis spectra of (a) Fc-COOH, (b) CNOs, (c) CNO 2, (d) CNO 4, (e) CNO 6, (f) CNO 8 and (g) CNO 10 in ethanol.

The CNO-Fc derivatives start to decompose in the temperature range between 100 and 176 °C. The most intense peaks are observed at 506, 524, 524, 500 and 400 °C for CNO 2, CNO 4, CNO 6, CNO 8 and CNO 10, respectively. The sharp mass-loss transition corresponding to the combustion of CNOs shows that the material is a homogeneous single phase with very few impurities. Fig. 4a reveals that CNO 2 and CNO 10 have the highest degree of CNO surface functionalization (Table 2).

### Photophysical properties of CNO-Fc derivatives

The UV-vis absorption spectra of the CNO-Fc derivatives and their reference compounds in ethanol solution are shown in Fig. 4. The UV-vis spectrum of the unmodified CNOs (Fig. 4b) shows no absorption peaks in the visible region but contains a wide absorption peak with two maxima at 286 and 291 nm consistent with previous reports.^42–45^ Characteristic absorption bands of Fc-COOH are observed in the regions of 260–280, 290–370 nm and 390–490 nm. The first band is ascribed to the π–π* transitions and metal-to-ligand charge transfer of the ferrocene, while the latter bands are assigned to the n–π* transitions of the carboxylic groups.^46,47^ A shoulder with high intensity at approximately 200 nm is derived from the ligand-to-metal charge transfer transitions from the π levels e_1g_(π), e_1u_(π) and e_1u_(π) to e_1g_(d).^46^ CNO 2, CNO 4, CNO 8 and CNO 10 show a broad absorption band in the UV from 240 nm to 320 nm. Only CNO 6 exhibits a narrow peak at ∼300 nm. The characteristic *Soret* band in the region of 250–400 nm is assigned to the π–π* transition of the aromatic group and to the metal-to-ligand charge transfer (e_2g_ → e_1u_) of ferrocene.^46,48^ The shapes of the broad bands for the CNO-Fc derivatives are different from those of the precursors, likely due to ground-state interactions between the ferrocene group and the multi-layered fullerene cages.^49^ The absorption bands of the CNO derivatives are shifted to shorter wavelengths, indicating that the CNOs are electron withdrawing, similar to C_60_ or MWNTs.

**Fig. 5:**
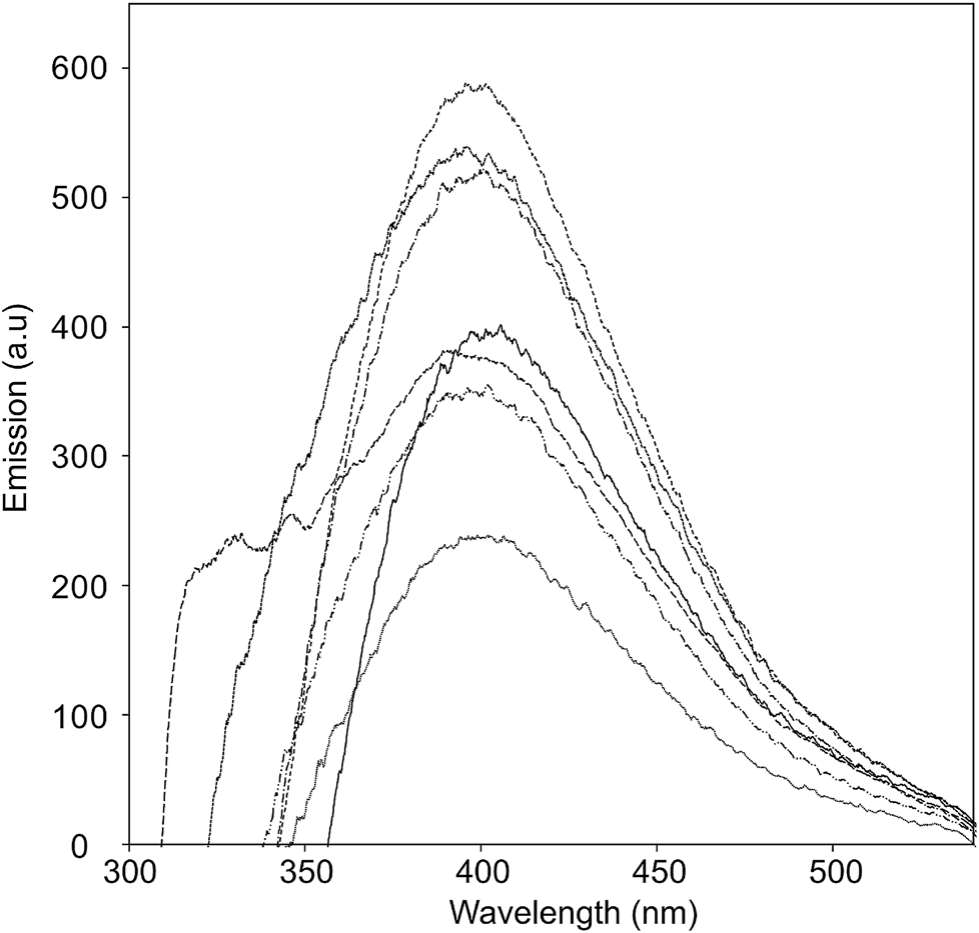
Fluorescence emission spectra of Fc-COOH (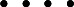), unmodified CNOs (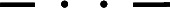), CNO 2 (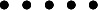), CNO 4 (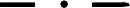), CNO 6 (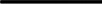), CNO 8 (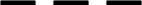), and CNO 10 (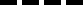) in ethanol (photoexcited at 280 nm).

The excited-state properties were examined by steady-state fluorescence measurements to probe the excitation of the donors. Fluorescence spectra were obtained for CNO 2, CNO 4, CNO 6, CNO 8 and CNO 10 (Fig. 5). Since the highest emission of Fc-COOH was observed using 300 nm excitation, the emission spectra of the CNO-Fc derivatives were obtained using this excitation wavelength. The emission intensities of the CNO-Fc derivatives increase in the following order: CNO 6 < CNO 4 < CNO 10 < CNO 8 < CNO 2. These intensities were all higher than that of pristine CNOs. The probability of excited state deactivation by fluorescence for each CNO-Fc derivative was measured and is expressed as the quantum yield, *ϕ*_f_ (Table 3).

**Table 3:** Optical and electrochemical data of CNO-Fc derivatives obtained by UV-vis spectroscopy, steady-state fluorescence and electrochemical measurements^a^

Sample	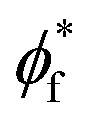	*E*_ox_ (onset) CV *vs.* Ag/AgCl	*E*_ox_ (max) DPV *vs.* Ag/AgCl	*λ*_onset_ (nm)	HOMO_CV_ (eV)	LUMO_opt_ (eV)	*E*_g_ (eV)
Fc-COOH	0.27	0.328	0.435	—	—	—	—
CNO 2	0.74	0.288	0.385	321	−5.00	−1.14	**3.86**
CNO 4	0.52	0.327	0.425	322	−5.04	−1.19	**3.85**
CNO 6	0.22	0.320	0.420	304	−5.03	−0.95	**4.08**
CNO 8	0.62	0.322	0.425	324	−5.03	−1.20	**3.83**
CNO 10	0.59	0.180	0.635	321	−4.89	−1.03	**3.86**

a *ϕ*_f_ quantum yields determined for Fc-COOH and CNO-Fc derivatives in ethanol; 
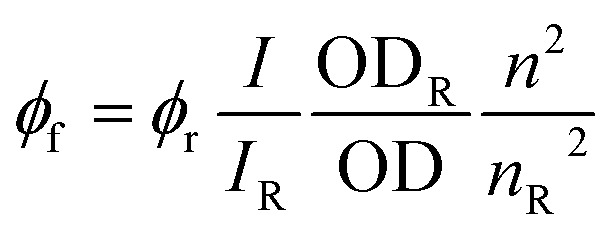
, *I* – integrated fluorescence intensity, OD – optical density, and *n* – refractive index; subscript ‘r’ refers to the reference fluorophore.

The highest quantum yield was observed for CNO 2, and the lowest for CNO 6. On the basis of the *ϕ*_f_ values and the UV-vis absorption spectra, it is concluded that the introduction of the best chromophore does not lead to the highest *ϕ*_f_ (Table 3). Therefore, the fact that the functionalization of CNOs may proceed with different yields is a consequence of not only different synthetic routes but also steric effects between donor groups on the surface.

Diffuse reflectance spectroscopy (DRS) was used to monitor the reflectance of the CNO and CNO:P3HT layers in solid phase (Fig. SI1†). This method is used for the characterization of chromophores, which are too opaque to permit the conventional use of UV-vis spectroscopy.^50,51^ The CNO and CNO:P3HT layers were studied by DRS in the range between 280 and 850 nm (Fig. SI1†). For both layers we observed interaction of light with the CNOs and CNOs:P3HT up to 850 nm (Fig. SI1†). Apparently, the reflectance of the chromophores has a different characteristics in solution and solid phase.^50,52^ Usually, if the physical adsorption (van der Waals interaction) occurs, the following differences between transmittance and reflectance are observed: the reflectance spectrum is broadened, the bands are displayed toward longer wavelengths, the vibrational structures is strongly suppressed, frequently appearing as a broad humps.^52^ Additionally, the active layers are very sensitive to every parameters during the film formation (temperature, solvent annealing, the weight ratio of components, aggregation of nanoparticles, thickness of the active blend layer, *etc.*).^53^ All the complementary features of the absorption bands of the CNO and CNO:P3HT layers broaden the light-harvesting wavelength range of these blends in their solid phase (Fig. SI1†).

### Electrochemistry of CNO-Fc derivatives

To estimate the energies of the HOMO and LUMO levels of the compounds, electrochemical characterizations were performed. The electrochemical studies of the CNO-Fc derivatives (CNO 2, CNO 4, CNO 6, CNO 8 and CNO 10) and of their reference compounds (CNO 1, CNO 3, CNO 5, CNO 7 and CNO 9) were carried out using both cyclic voltammetry (CV) and differential pulse voltammetry (DPV).

**Fig. 6:**
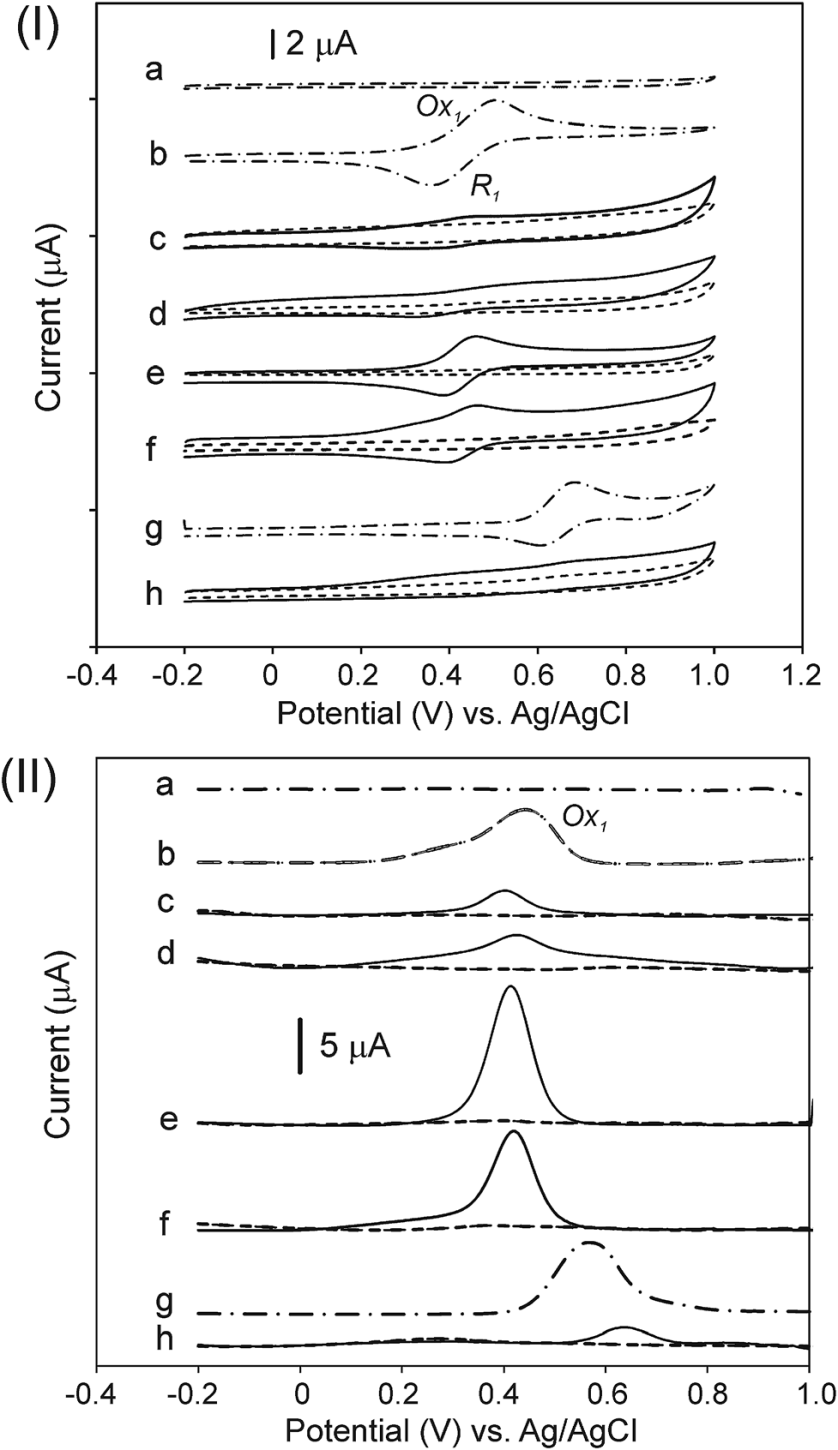
(I) CV and (II) DPV of the CNO-Fc derivatives: (a) CNO, (b) Fc-COOH, (c) CNO 1 and CNO 2, (d) CNO 3 and CNO 4, (e) CNO 5 and CNO 6, (f) CNO 7 and CNO 8, (g) Fc-P, and (h) CNO 9 and CNO 10. The measurements were performed in an ACN/tol mixture (1 : 4, v/v) with 0.1 mol L^−1^ TBAPF_6_ at 10 mV s^−1^. The CNO derivatives (CNO 1, CNO 3, CNO 5, CNO 7, and CNO 9) are marked as dashed lines, and the ferrocene systems (CNO 2, CNO 4, CNO 6, CNO 8, and CNO 10) are marked as solid lines.

Fig. 6 shows the CV (Fig. 6, panel (I)) and DPV (Fig. 6, panel (II)) plots for the compounds. In addition, Table 3 summarizes the measured potentials as determined by CV and DPV. The measurements were performed in an acetonitrile/toluene (ACN/tol) mixture (1 : 4, v/v) with 0.1 mol L^−1^ TBAPF_6_, as the supporting electrolyte, at 25 °C and at a scan rate 50 mV s^−1^ under an argon atmosphere. All products originating from the CNO-Fc derivatives and their ferrocene reference compounds show electrochemically reversible first oxidation waves (Fig. 6, panel (I)). The Ox_1_ and R_1_ peaks observed for the CNO-Fc systems (CNO 2, CNO 4, CNO 6, CNO 8 and CNO 10) correspond to the reversible one-electron oxidation-reduction of iron^67^ and to diffusion-controlled Fc/Fc^+^ oxidation.^54,55^

The compounds containing ferrocenyl groups show a one-electron oxidation process at potentials that vary little with the spacer group between the CNO core and the Fc moiety.^54^ Furthermore, the redox properties of hybrids CNO 2, CNO 4, CNO 6, CNO 8 and CNO 10 are different from those of the model compounds CNO 1, CNO 3, CNO 5, CNO 7 and CNO 9, respectively. When comparing the behaviour of Fc-COOH to that of CNO 2, CNO 4, CNO 6 and CNO 8, the oxidation potential clearly shifts from +0.44 V to more negative potentials, reflecting the acceptor property of the CNOs (Fig. 6, panel (II)). The scan of CNO 10 is different from that of the other ferrocene derivatives, as shown in Fig. 6h. DPV shows that the oxidation peak is shifted anodically to +0.64 V (*vs.* Ag/AgCl) compared to the +0.54 V of the Fc-P model.

The electronic/optical energy level (*E*_g_) is a key parameter of conjugated photosensitive structures. *E*_g_ values can be calculated from the electrochemical measurements or from the absorption spectra, by subtracting the highest occupied molecular orbital (HOMO) and lowest unoccupied molecular orbital (LUMO) energies.

The HOMO energy levels of the CNO-Fc derivatives were estimated from the onset oxidation potential of the first peak (*E*_ox(onset)_) obtained from the CVs, based on eqn (1):

1 HOMO = −(*E*_ox(onset)_ + *C*) (eV)

where *E*_ox(onset)_ is the potential *versus* the reference electrode (RE) and *C* is a constant related to the RE used.^65^ If the RE is Ag/Ag^+^, then *C* takes the value of 4.71.^65^ The LUMO energy levels can be calculated from the values of the HOMO and the optical band gaps determined at the onset of absorption from UV-vis measurement:^66,67^

2 LUMO = HOMO + *E*_g_ (eV)

3
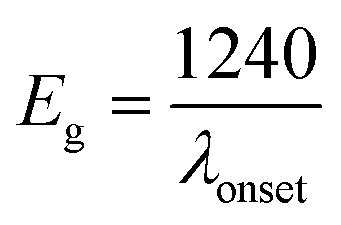


The calculated HOMO and LUMO energy levels are collected in Table 3. The electrochemical HOMO energy levels (calculated using eqn (1)) of the CNO-Fc derivatives are between −4.89 eV (CNO 10) and −5.04 eV (CNO 4) (Table 3). The optical LUMO energy levels (calculated using eqn (2) and (3)) of the CNO-Fc derivatives are between −0.95 eV (CNO 6) and −1.20 eV (CNO 8). The HOMO energy levels of the CNO-Fc derivatives are dominated by the ferrocene group, and the LUMO energy levels of the CNO-Fc derivatives are centered on the carbon cages. Thus similar band gaps of ∼3.85 eV are obtained for CNO 2, CNO 4, CNO 8 and CNO 10. The lowest value of *E*_g_ = 3.85 eV and the highest value of *E*_g_ = 4.08 eV are obtained for CNO 4 and CNO 6, respectively, and are consistent with the results obtained from the steady-state fluorescence measurements (Table 3).

### Textural properties of CNO-Fc derivatives

The porosity, pore size distribution, and specific surface area were characterized by nitrogen sorption isotherms at 77 K. All types of CNOs were analysed by the multilayer model of adsorption and the Brunauer–Emmett–Teller (BET) static nitrogen adsorption technique.^68^ The functionalization of the CNO cages with ferrocene leads to significant transformations of the CNOs' textural parameters. As revealed, the functionalization process of CNOs results in remarkable changes to N_2_ adsorption, which is indicative of a decrease in the specific surface area (*S*_BET_), external surface area (*S*_ext_) (without a microporous structure for CNO 6), cumulative volume of pores and microporosity, and increase in average pore width (Table SI1†). The total surface area of the micropores increases from 46 m^2^ g^−1^ (unmodified CNOs) to 99 m^2^ g^−1^ (CNO 6), with the highest values being obtained for the Fc-CNOs. The same tendency is observed for the micropore volume of the micropores, from 0.0195 cm^3^ g^−1^ (unmodified CNOs) to 0.0389 cm^3^ g^−1^ (CNO 6). Barrett, Joyner, and Halenda (BJH) proposed a method which enables the calculation of pore diameters (*d*_p_) as a function of the pore volume (d*V*_p_/d*d*_p_) and of the surface area (d*S*/d*d*_p_), according to the formula:^68^4
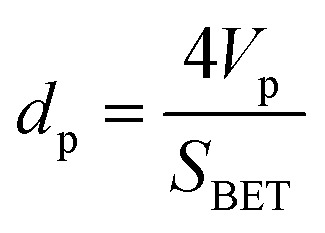
where *d*_p_ is the average pore width (nm), *V*_p_ is the pore volume (cm^3^ g^−1^) and *S*_BET_ is the specific surface area (m^2^ g^−1^). With the functionalization of CNOs, the pore volume, *V*_p_, decreases from 1.660 to 0.360 cm^3^ g^−1^ for CNOs and CNO 5, respectively. The highest value of the pore volume (0.927 cm^3^ g^−1^) is observed for CNO 4. The average pore width was also estimated from the pore volume, and it is observed that the average pore width of ∼12 nm did change significantly, even up to 21 nm for CNO 4. Thus, as anticipated, functionalization greatly influences the porous structures of carbon materials.

### Photovoltaic performances of CNO-Fc derivatives

The photovoltaic properties of CNO-Fc derivatives were investigated in bulk heterojunction (BHJ) OPV cells. Devices with an inverted architecture of ITO-coated glass/ZnO/P3HT:CNO-Fc/Ag were used. BHJ solar cells can be made *via* solution processing, spin-casting or printing.^69,70^ One of the most important parameters that directly impacts the efficiency of the device is the morphology and composition of the photoactive layer. To increase the crystallinity of P3HT we used *o*-DCB and an annealing protocol.^71,72^ Additionally, the performance of OPV devices have been improved by using a ZnO layer, which serves as the electron transporting layer (ETL).^73^

The morphology and interfacial behaviour of the multicomponent active layer confined between the electrodes are influenced by the preparation conditions and solubilities of the photoactive compounds.^74^ The morphology and roughness of each layer of the OPV cell were studied by scanning electron microscopy (SEM) for CNO 4 as part of the active layer (Fig. SI2†). The SEM images at different magnifications of the ZnO layer are shown in Fig. SI2a–c.† The surface of ZnO is perfectly flat and homogeneous, and consequently, it is a great foundation for the active layer of the OPV (Fig. SI2†). The morphology of the P3HT:CNO 4 layer (Fig. SI2d–f†) differs significantly from the morphology of thin films of ZnO (Fig. SI2a–c†) due to unevenness of the surface, which results from the nature of CNOs and their aggregation properties. Nevertheless, the dispersibility of CNOs in polar and non-polar solvents also strongly depends on their functionalization. Homogenous distributions of CNO particles are obtained for all CNO derivatives except for CNO 6, which shows low dispersibility in *o*-DCB.

For all OPV devices we used the same mass concentration of the CNO derivatives (8 mg mL^−1^ in a P3HT:CNO-Fc dispersion). The CNOs and their derivatives have a limited dispersibility in all solvents. The nitrogen adsorption studies indicated that CNO 4 had the highest value of the pore volume and additionally showed the highest dispersion in *o*-DCB. These two factors should have a significant positive impact on the OPV performance parameters. The extended absorption of sunlight at longer wavelengths directly reflects on the value of *J*_sc_,^75,76^ and a higher current density was achieved (Fig. SI3† and Table 4).

**Table 4:** Parameter details of the OPV devices based on CNOs-Fc

Acceptor	*J*_sc_ a (mA cm^−2^)	*V*_oc_ (V)	FF^b^ (%)	PCE (%)
CNOs	3.07	0.08	22	**0.05**
CNO 2	7.51	0.17	40	**0.51**
CNO 4	10.66	0.35	49	**1.89**
CNO 6	3.67	0.09	23	**0.08**
CNO 8	6.80	0.14	35	**0.33**
CNO 10	7.85	0.23	40	**0.72**

a The calculated short circuit current values were obtained from the external quantum efficiency curves (Fig. SI3) using the correlation 
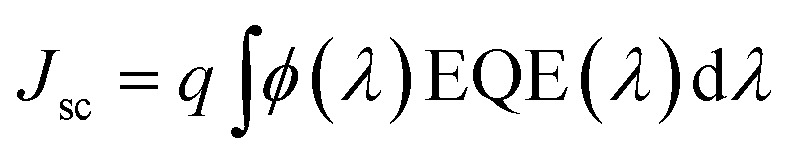
.

b The calculated FF were obtained using equation FF = *P*_max_/*V*_oc_*J*_sc_ with *J*_sc_ calculated as shown.^a^

**Fig. 7:**
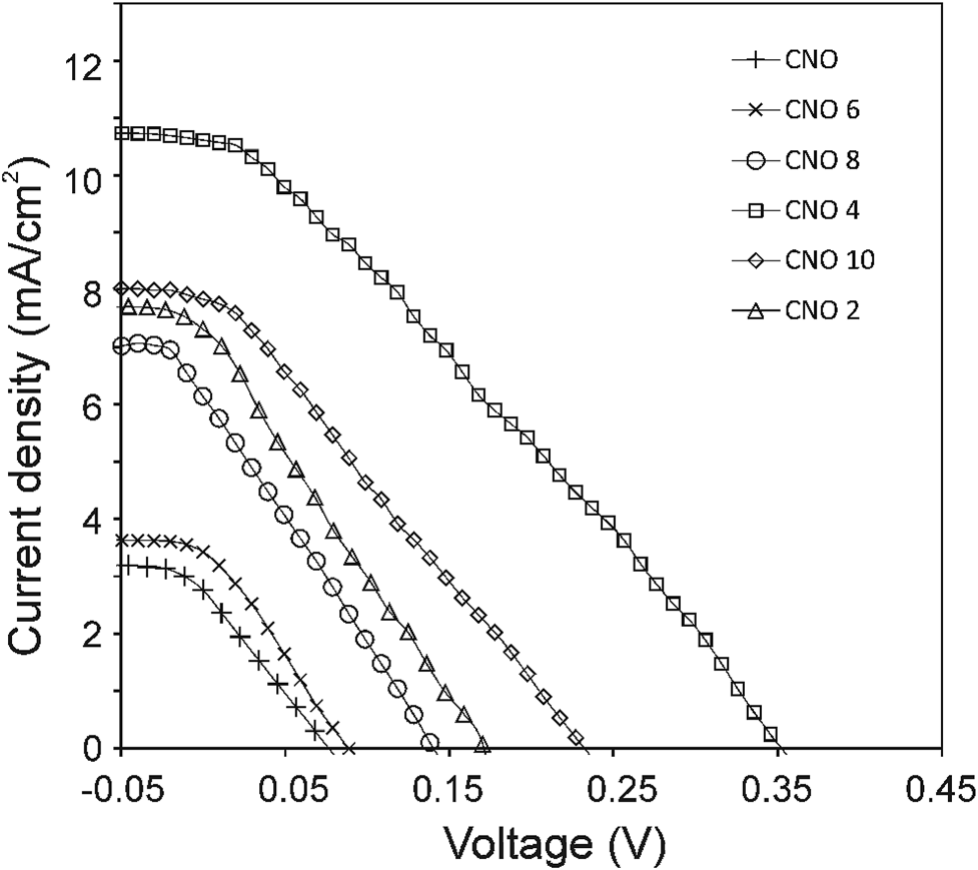
*J*–*V* characteristics of the OPV devices with CNOs-Fc in the active layer.

The current density–voltage (*I*–*V*) characteristics of all CNO derivatives used as components of the active layer in OPV devices are shown in Fig. 7. All measurements were done under illumination equal to AM 1.5 G (100 mW cm^−2^) at a current density in the range between *ca.* 3 and 11 mA cm^−2^. Using the measured external quantum efficiency (EQE) data *J*_sc_ was calculated under 100 mW cm^−2^ of AM 1.5 G solar irradiation, according to the formula:5
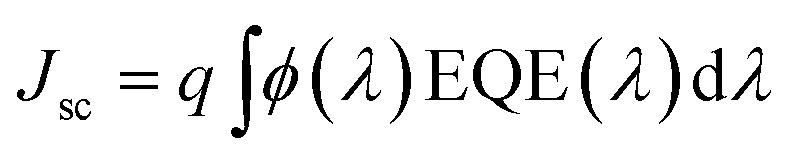
where *q* is the charge of the electron, and *ϕ* is the photon flux. The photovoltaic parameters for these devices are summarized in Table 4 and represent the measured average values.

The highest PCE equalling 1.89% was obtained with an open-circuit voltage of 0.46 V (*V*_oc_), a *J*_sc_ of 10.66 mA cm^−2^ (calculated from the EQE curves, Fig. SI3†) and a fill factor of 49% (FF) for the CNO 4-based device. The *J*_sc_ of the BHJ solar cells for all five CNO-Fc derivatives range from 3.67 to 10.66 mA per cm^2^ and the FF values from 23 to 49%. The device constructed with non-modified CNO shows a PCE value of 0.05% (*J*_sc_ = 3.07 mA per cm^2^, *V*_oc_ = 0.09 V, and FF = 22%). The lowest PCE value (0.08%), *V*_oc_ (0.10 V), and FF (23%) was observed for the device in which P3HT:CNO 6 is used as the active layer. These values are strongly correlated with the high aggregation of this derivative in solution. Experimental evidence shows, that the PCE of these devices is improved by functionalization of the CNOs, which decreased the CNO's *E*_g_ and increased their dispersibility.

However, the obtained PCE results are still far from the values of other OPV devices with incorporated CNs, where these nanostructures were used as a light harvester or as charge transporters (Table 5). It has to be underlined that CNOs are used as an acceptor' in our OPV cells.

**Table 5:** Photovoltaic data of representative devices from the literature in which CNs have been used in the active layer^a^

Devices	PCE (%)	Note	Ref.
**Pristine without dopants**			
ITO/PEDOT/SWNT:P3HT/BCP/Al	0.72	Using semiconducting SWNTs coated with P3HT	56
ITO/ZnO/P3HT:SWNT/MoS_2_/PEDOT/Au	0.46	SWNTs enabled a mixture of MoS_2_ and P3HT	57
ITO/PEDOT/CNT:P3HT:PCBM/LiF/Al	2.00	—	58
ITO/PDDA/SWNT–PVBTA^*n*+^/ZnP^8−^	3.81–9.90	For covalent and non-covalent functionalization of SWNTs with PVBTA	59
ITO/ZnO/CNO 4:P3HT/Ag	**1.89**	—	**This work**

**With dopants**			
ITO/PEDOT/SWNT/C_60_/Ag	0.46	—	60
ITO/PEDOT:PSS/MWNT:C_60_/Al	0.80	—	61
ITO/PEDOT/PTB7:PC_71_BM:N-CNT/Ca/Al	8.60	—	62
ITO/PEDOT/P3HT:ICBA:QD:N-CNT/TiO_*x*_/Al	6.11	QDs were used to enhance doping and dispersion	63
ITO/PEDOT/P3HT:PCBM:B-MWNT/TiO_*x*_/Al	4.10	B-MWNTs were used to enhance PCE	64
ITO/PEDOT:PSS:CNO/perovskite/PCBM/Bphen/Ag	15.26	—	32

a Abbreviations: PEDOT – poly(3,4-ethylenedioxythiophene); BCP – bathocuproine; PDDA – poly(diallyldimethylammonium chloride); P3HT – poly(3-hexylthiophene); PCBM – [6,6]-phenyl-C_61_-butyric acid methyl ester; PVBTA^*n*+^ – poly[(vinylbenzyl)trimethylammonium chloride]; ZnP^8−^ – Zn porphyrins; PTB7 – polythieno[3,4-*b*]thiophene/benzodithiophene; PC_71_BM – [6,6]-phenyl-C_71_-butyric acid methyl ester; N-CNT – nitrogen-doped carbon nanotubes; ICBA – indene-C_60_ bisadduct; QD – quantum dots; B-MWNT – boron-doped multi-walled carbon nanotubes; Bphen – bathophenanthroline.

Several examples of OPV devices containing CNs as dopants are presented in Table 5. Systems similar to our OPV devices, with single-walled carbon nanotubes incorporated in an active layer, provided a PCE of *ca.* 1% (Table 5). One of the highest PCE values was obtained by Prato and co-workers for the SWNT–poly[(vinylbenzyl)trimethylammonium chloride] (PVBTA^*n*+^).^59^ SWNT–PVBTA^*n*+^ was synthesized by the free-radical polymerization of (vinylbenzyl)trimethylammonium chloride. PVBTA^*n*+^ was also noncovalently wrapped around SWNTs to form a positively charged SWNT/PVBTA^*n*+^ suspension in water. Photocurrent measurements gave PCEs of 3.81 and 9.90% for the covalently and noncovalently modified SWNTs, respectively. It was already stressed that one of the highest PCE values for OPV devices was obtained for ITO/PEDOT:PSS:CNOs/perovskite/PCBM/Bphen/Ag (Table 5).^32^ In this system, spherical CNOs were used as dopants in PEDOT:PSS, which reduced the acidity and hydrophilicity of this layer and suppressed the corrosion of ITO.

This comparison shows that the use of larger CNs in OPV devices is also very promising. It should be emphasized that the CNO-Fc derivatives were used to construct one of the simplest OPV systems reported in the literature.

## Experimental

### Materials and methods

All chemicals and solvents were used without additional purification: thionyl chloride (≥99%, Sigma-Aldrich, Poland), ammonia solution (25% pure p.a, Sigma-Aldrich), 4-aminobenzylamine (∼99%, Sigma-Aldrich), *o*-anisidine (≥99%, Sigma-Aldrich), phenol (≥99%, Sigma-Aldrich), formaldehyde (36–38% pure p.a, Sigma-Aldrich), maleimide (∼99%, Sigma-Aldrich), 1,2-dicyanobenzene (∼98%, Sigma-Aldrich), zinc chloride (≥98%, Sigma-Aldrich), isopentyl nitrite (∼96%, Sigma-Aldrich), ferrocenecarboxylic acid (≥97%, Sigma-Aldrich), 1,1′-bis(diphenylphosphino)-ferrocene (≥97%, Sigma-Aldrich), ethanol (∼96%, pure p.a., POCH, Poland), tetrabutylammonium hexafluorophosphate (≥98%, Sigma-Aldrich), acetone (99.5% pure p.a, POCH), toluene (≥99.7% pure p.a, Sigma-Aldrich), nitric acid (∼65%, pure p.a, POCH), hydrochloric acid (35–38%, pure p.a, POCH), acetonitrile (99.8%, Sigma-Aldrich), dimethylformamide (99.9+%, Sigma-Aldrich), benzene-d_6_ (∼99.5%), regioregular poly(3-hexylthiophene-2,5-diyl) (P3HT, Sigma-Aldrich), zinc oxide dispersion (nanoparticles, 40 wt% in ethanol, <130 nm particle size; Sigma-Aldrich), and *o*-dichlorobenzene (Sigma-Aldrich). All aqueous solutions for electrochemical studies were made using deionized water, which was further purified with a Milli-Q system (Millipore).

FTIR-spectra were recorded with an FT-IR Nicolet iN10 MX microscope in the range from 4000 to 700 cm^−1^ using an array detector cooled by liquid nitrogen. The ^1^H NMR spectra were recorded on a Bruker Avance II spectrometer (400 MHz) at room temperature in deuterobenzene and were referenced to residual solvent peaks. The chemical shifts are represented in ppm. The Raman spectra were collected using a Renishaw spectrometer with a liquid N_2_-cooled charge-coupled device (CCD) within a range from 3500–100 cm^−1^ excited by an Ar^+^ ion laser (514 nm) and a 20× lens. SEM images were recorded with an SEM FEI Tecnai S-3000N microscope 1000× with a resolution of 100 μm. The samples were applied to gold foil and prepared on an aluminium plate.

UV-vis spectra were recorded using a Hitachi U-3900H spectrophotometer equipped with a double monochromator and a double-beam optical system (190–800 nm). Steady-state emission spectra were recorded on a Hitachi F7000 fluorescence spectrophotometer. The measurements were carried out at room temperature. The diffuse reflectance (DR) measurements were made by an elipsometric spectrophotometer SENTECH Instruments GmbH SE850. The spectra were registered in the spectral range between 280–850 nm.

Thermogravimetric experiments were performed using an SDT 2960 with simultaneous TGA-DTG (TA Instruments company). The thermogravimetric traces were recorded at 15 °C min^−1^ in an air (100 mL min^−1^).

N_2_ gas adsorption measurements were performed using a Micromeritics apparatus (ASAP2020 – automatic sorption analyser, Micromeritics Corp., USA) at −196 °C. Prior to the gas adsorption analysis, all samples were degassed at 100 °C and 10 μm Hg vacuum for 20 h to remove any adsorbed species.

Voltammetric experiments were performed using an AUTOLAB (Utrecht, The Netherlands) computerized electrochemistry system equipped with a PGSTAT 12 potentiostat using a three-electrode cell placed in a Faraday cage. The AUTOLAB system was controlled with the Nova 1.10 software from the same manufacturer. A glassy-carbon disk electrode (Bioanalytical Systems, Inc.) with a diameter of 2 mm (Bioanalytical Systems, Inc.) was used as the working electrode. The surface of the electrode was polished using extra-fine carborundum paper (Buehler), followed by 0.3 μm alumina and 0.25 μm diamond polishing compounds (Metadi II, Buehler). The electrode was then sonicated in water to remove traces of alumina from the metal surface, washed with water, and dried. The counter electrode was a platinum square with an area of approximately 0.5 cm^2^. A silver wire immersed in 0.01 mol L^−1^ AgCl and 0.09 M TBAClO_4_ and separated from the working electrode by a ceramic tip (Bioanalytical Systems, Inc.) served as the reference electrode. The active material (CNO 1–CNO 10 and their ferrocene references) with a concentration 0.5 mg mL^−1^ was suspended in an acetonitrile and toluene mixture at a volume ratio 1 : 4 with 0.1 mol L^−1^ TBAPF_6_ as the supporting electrolyte. Oxygen was removed from the solution by purging with argon.

The *J*–*V* curves were measured using solar simulator Model #SS50AAA PET from Photo Emission Tech, Inc. with a Keithley 2401 source-meter. The lamp emitted standardized sunlight, whose spectrum of radiation was close to a spectrum of an air mass 1.5 global AM (1.5 G) irradiation. Additionally, an atmospheric correction was used, assuming that all measurements were made at 25 °C and at a standard radiation intensity of 100 mW cm^−2^. The solar simulator was calibrated using a reference silicon solar cell.

### Device preparation

In this paper, OPV devices were fabricated with the configuration: ITO-coated glass/ZnO/P3HT:CNOs-Fc/Ag. In the beginning, ITO-coated glass was cleaned by sonication in water with detergent, water, acetone and isopropanol for 30 min each. After drying, 150 μL ZnO was spin-coated onto the ITO at 2000 rpm and annealed on a hot plate at 180 °C for 20 min. The photoactive materials were P3HT as the electron donor and an CNO-Fc derivative as the acceptor. The active layer consisted of P3HT and CNO-Fc blended in a mass ratio of 3 : 2, which were previously dissolved in *o*-dichlorobenzene (*o*-DCB) and mixed for 24 h at 50 °C under an ambient atmosphere. *o*-DCB was chosen because of its good solvent properties and low evaporation rate.^77^ The active layer was prepared by spin-casting films from an *o*-DCB solution comprising P3HT:CNO-Fc (20 mg mL^−1^). Devices were annealed on a hot plate in an Ar glovebox at 80 °C for 20 min. The metal electrode was Ag and was evaporated from a silver target in a specialist sputter machine under vacuum. Two silver electrodes were sputtered in shadow mask. The effective area of the cell was *ca.* 0.02 cm^2^. The solar cells were not encapsulated.

### Synthetic procedures

#### General procedure for the functionalization of CNOs

##### Synthesis of CNO 1

Oxidized-CNOs (ox-CNOs) were obtained by the oxidation of CNOs in 3 mol dm^−3^ HNO_3_ according to already described procedures. ox-CNOs (50 mg) were dispersed in 5 mL DMF, and then, 30 mg SOCl_2_ and 5 mg ZnCl_2_ were added carefully. The reaction was stirred for 6 h at 70 °C. Subsequently, 0.1 mL 25% aqueous ammonia solution was added, and the reaction was stirred for 24 h at 110 °C. The mixture was washed with deionized water and acetone. The product CNO 1 was dried at 50 °C overnight. ^1^H NMR (400 MHz, C_6_D_6_, *δ*, ppm): 5.31, 4.21, 3.76, 3.5, 3.0, 2.36, 2.18, 2.15, 1.86, 1.6, 1.3, 0.87. FTIR (cm^−1^): 3422, 2701, 1878, 1615, 1409, 1294, 1128, 1052, 987, 865.

##### Synthesis of CNO 2

CNO 1 (40 mg) and 28 mg ferrocene carboxylic acid were added into a 50 mL one-necked round-bottom flask with 6 mL DMF. This mixture was stirred at 90 °C for ∼24 h. The reaction was stopped, and the residue was washed repeatedly with water and acetone. The mixture was evaporated under reduced pressure, and then, the product was dried in an oven at 50 °C. ^1^H NMR (400 MHz, C_6_D_6_, *δ*, ppm): 7.22, 5.35, 4.14, 4.03, 3.76, 3.50, 2.18, 2.05, 1.86, 1.58, 1.27, 0.89. FTIR (cm^−1^): 3172, 1812, 1548, 1435, 1189, 1001, 839.

##### Synthesis of CNO 3

CNOs (50 mg) and 24.6 mg 4-aminobenzylamine were added into a 50 mL one-necked round-bottom flask, and then, 20 mg isopentyl nitrite, as a catalyst, was added dropwise by a syringe. The reaction was allowed to proceed for ∼24 h at 60 °C. The product was purified by washing with acetone and dried overnight at 50 °C. ^1^H NMR (400 MHz, C_6_D_6_, *δ*, ppm): 7.28, 5.35, 4.21, 4.03, 3.75, 3.50, 2.36, 2.18, 2.05, 1.83, 1.56, 1.27, 0.85. FTIR (cm^−1^): 3389, 2869, 1908, 1600, 1526, 1298, 1042, 913.

##### Synthesis of CNO 4

CNO 3 (40 mg) was suspended in 6 mL of dimethylformamide, and 22 mg ferrocenecarboxylic acid was added into a round-bottom flask. The mixture was heated for ∼24 h at 90 °C. The residue was washed with water and acetone and then dried in an oven at 50 °C. ^1^H NMR (400 MHz, C_6_D_6_, *δ*, ppm): 7.25, 6.29, 5.32, 4.16, 4.03, 3.76, 3.50, 2.46, 2.36, 2.18, 2.02, 1.86, 1.61, 1.26, 0.90. FTIR (cm^−1^): 3356, 2926, 2006, 1834, 1659, 1342, 1024, 852.

##### Synthesis of CNO 5

CNO 5 was synthesized in two steps. 1,2-Dicyanobenzene (34 mg) was first reacted with phenol (25 mg) without any solvent for 6 h at 130 °C. Next, 65 mg of pristine CNOs was suspended in 5 mL of toluene and added to the reaction mixture. The reaction was allowed to proceed for ∼24 h at 100 °C. The residue was washed with water and acetone. The product was dried at 50 °C overnight. ^1^H NMR (400 MHz, C_6_D_6_, *δ*, ppm): 7.2, 4.26, 3.01, 2.37, 1.86, 1.55, 1.32, 0.92. FTIR (cm^−1^): 3437, 2948, 2005, 1877, 1508, 1247, 1002, 853.

##### Synthesis of CNO 6

CNOs 5 (40 mg) and 20 mg of ferrocenecarboxylic acid were added into a 50 mL one-necked round-bottom flask with 5 mL DMF. The reaction was stirred for ∼24 h at 90 °C. The product was purified by washing with acetone and dried at 50 °C overnight. ^1^H NMR (400 MHz, C_6_D_6_, *δ*, ppm): 7.2, 4.25, 3.58, 3.00, 2.11, 1.54, 1.40, 1.24, 0.95, 0.87. FTIR (cm^−1^): 3392, 2921, 2018, 1669, 1605, 1498, 1436, 1330, 1105, 999, 868.

##### Synthesis of CNO 7

CNOs (50 mg) and maleimide (24.5 mg) were added into a 50 mL one-necked round-bottom flask with 5 mL DMF. The resulting mixture was refluxed for ∼24 h in the presence of zinc chloride (5 mg). Next, the mixture was washed with deionized water and acetone. The product was dried at 50 °C overnight. ^1^H NMR (400 MHz, C_6_D_6_, *δ*, ppm): 7.2, 5.50, 4.26, 3.56, 2.32, 1.81, 1.62, 1.55, 1.34, 0.91. FTIR (cm^−1^): 3407, 2960, 2825, 2060, 1871, 1542, 1288, 990, 829.

##### Synthesis of CNO 8

CNO 7 (40 mg) and 24 mg ferrocene carboxylic acid were added into a round-bottom flask with 3 mL of DMF. The reaction was allowed to proceed for ∼24 h at 90 °C. After ending the reaction, the product was purified by washing with deionized water and acetone and dried in an oven at 50 °C. ^1^H NMR (400 MHz, C_6_D_6_, *δ*, ppm): 7.2, 4.26, 3.58, 3.02, 2.11, 1.54, 1.40, 1.38, 1.25, 0.96, 0.88. FTIR (cm^−1^): 2962, 2229, 1998, 1655, 1473, 1285, 997, 877, 834.

##### Synthesis of CNO 9

CNOs (40 mg) and 18.4 mg *o*-anisidine were added into a round-bottom flask. Next, 0.1 mL of isopentyl nitrite was added dropwise by a syringe. The reaction was stirred for ∼24 h at 60 °C. The product was purified by washing with acetone and drying at 50 °C overnight. ^1^H NMR (400 MHz, C_6_D_6_, *δ*, ppm): 7.25, 5.31, 4.26, 4.01, 3.50, 2.47, 2.35, 2.15, 1.84, 1.54, 1.25, 0.86. FTIR (cm^−1^): 2818, 2095, 1876, 1645, 1405, 1267, 975, 851.

##### Synthesis of CNO 10

CNO 9 (40 mg), 18 mg 1,1′-bis(diphenylphosphino)-ferrocene and 60 mL of formaldehyde were added into a round-bottom flask. The mixture was stirred for ∼24 h at 90 °C. After ending the reaction, the residue was washed repeatedly with water and acetone, and then, the product was dried in an oven at 50 °C. ^1^H NMR (400 MHz, C_6_D_6_, *δ*, ppm): 7.25, 5.28, 4.25, 4.01, 3.48, 2.47, 2.35, 2.17, 2.01, 1.84, 1.56, 1.25, 0.86. FTIR (cm^−1^): 3000, 2887, 2773, 1718, 1651, 1559, 1538, 1451, 1264, 1082, 865.

## Conclusions

Ferrocene-functionalized CNO systems were synthesized *via* the covalent functionalization of carbon nanoonion cages. These macromolecular structures possess unique physico-chemical properties. The absorption, fluorescence and electrochemical experiments showed that functionalization of the CNO cages with Fc moieties results in the formation of photo- and electroactive systems. These properties of CNO-Fc derivatives were confirmed using current-density–voltage characteristics. The CNOs-Fc were used as acceptors in the active layer of bulk heterojunction OPV cells. Notably, this report is the first, to the best of our knowledge, in which larger CNs without any traditional dopants (such as PCBM or perovskite) were used in the active layer of OPVs. This study reveals that the highest PCE conversion was obtained for the OPV device (1.89%) where the CNO 4 derivative was incorporated into the active layer of the OPV as an acceptor of electrons.

## Conflicts of interest

There are no conflict to declare.

## Supplementary Material

NA-001-C9NA00135B-s001
