# Supplementary material for: Carbon nanoonion-ferrocene conjugates as acceptors in organic photovoltaic devices
Source: Nanoscale Adv. 2019 Jul 3;1(8):3164–76. doi: 10.1039/c9na00135b (PMC9417719; doi:10.1039/c9na00135b)
Supplement: NA-001-C9NA00135B-s001 [file NA-001-C9NA00135B-s001.pdf]

Electronic Supplementary Material (ESI) for Nanoscale Advances.
This journal is © The Royal Society of Chemistry 2019

Supporting Information

Carbon Nanoonion-Ferrocene Conjugates as acceptors in organic photovoltaic devices

Diana M. Bobrowska,[a] Halyna Zubyk,[a] Elzbieta Regulska,[a] Elkin Romero,[b] Luis Echegoyen,[b] and Marta E. Plonska-Brzezinska[c]

[a] Institute of Chemistry, University of Bialystok, K. Ciolkowskiego 1K, 15-245 Bialystok, Poland.

[b] University of Texas at El Paso, 500 W University Ave., Chemistry and Computer Science Bldg. #2.0304, El Paso, TX 79968-8807, USA

[c] Department of Organic Chemistry, Faculty of Pharmacy with the Division of Laboratory Medicine, Medical University of Bialystok, Mickiewicza 2A, 15-222 Bialystok, Poland,
*E-mail: *marta.plonska-brzezinska@umb.edu.pl. Phone: (+4885) 748 5683.*

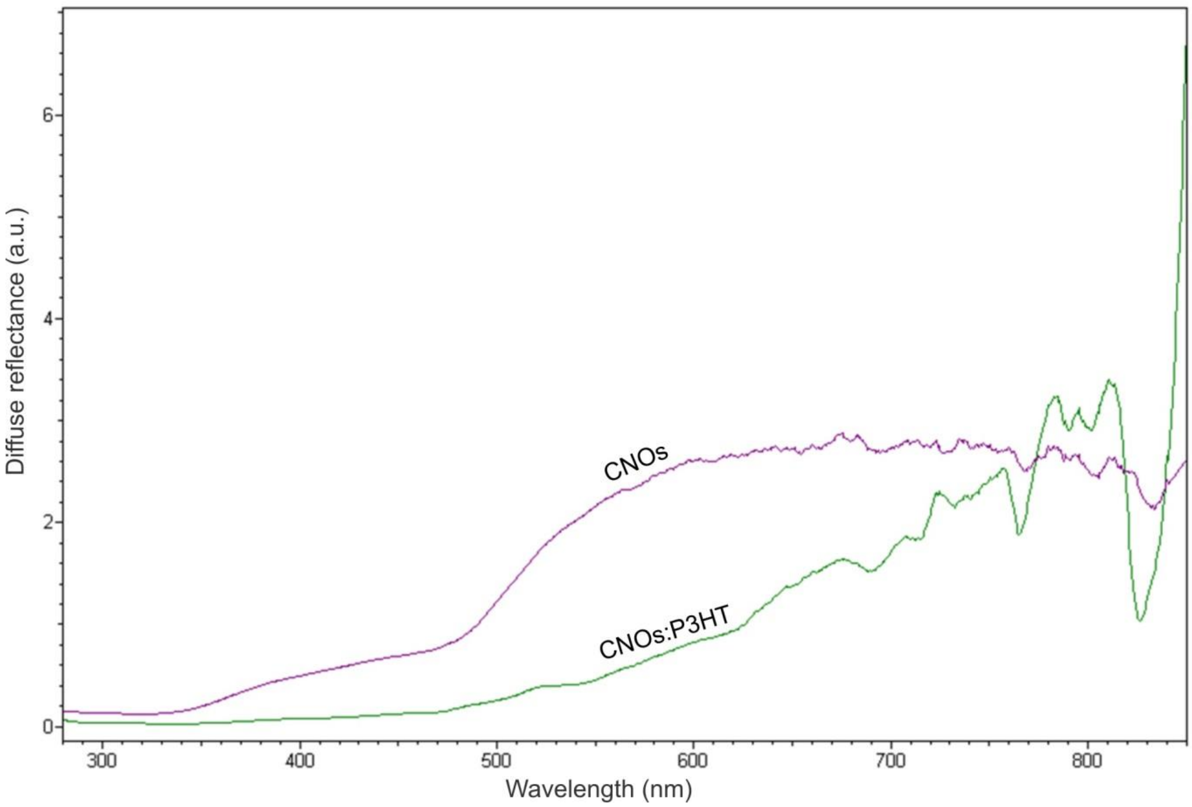

Fig. SI1. Diffuse reflectance spectra of the CNO and CNO:P3HT layers prepared by spin-coating method.

Table SI1. The physicochemical parameters of pristine and functionalized CNOs.

Sample	BET Surface Area S_{BET} (m^2/g)	t-plot micropore area (m^2/g)	t-Plot external surface area S_{ext} (m^2/g)	Cumulative surface area of pores [a] (m^2/g)	Cumulative surface area of pores [b] (m^2/g)	t-plot micropore volume (cm^3/g)	Cumulative volume of pores [a] (cm^3/g)	Cumulative volume of pores [b] (cm^3/g)	Average pore width, (nm) [c]	Average pore width, (nm) [d]
**CNOs**	454	46	408	505	547	0.0195	1.660[e]	1.686	13	12
**CNO 4**	201	42	159	177	195	0.0145	0.927[h]	0.884	21	18
**CNO 5**	108	42	67	85	110	0.0163	0.360[f]	0.375	17	14
**CNO 6**	214	99	116	183	200	0.0389	0.715[g]	0.724	16	15
**CNO 10**	148	19	129	144	154	0.0062	0.721[i]	0.684	20	18

[a] - based BJH Adsorption method (pores width between 17, 000 Å and 3000,000 Å)

[b] - based BJH Desorption method (pores width between 17, 000 Å and 3000,000 Å)

[c] - based BJH Adsorption method (4v/A), [d] - based BJH Desorption method (4v/A)

Single point adsorption total pore volume of pores less than: (e) 1390 Å diameter at p/p^o = 0.9862; (f) 1330 Å diameter at p/p^o = 0.9855; (g) 1379 Å diameter at p/p^o = 0,9861; (h) 1431 Å diameter at p/p^o = 0.9266; (i) 1307 Å diameter at p/p^o = 0.9853.

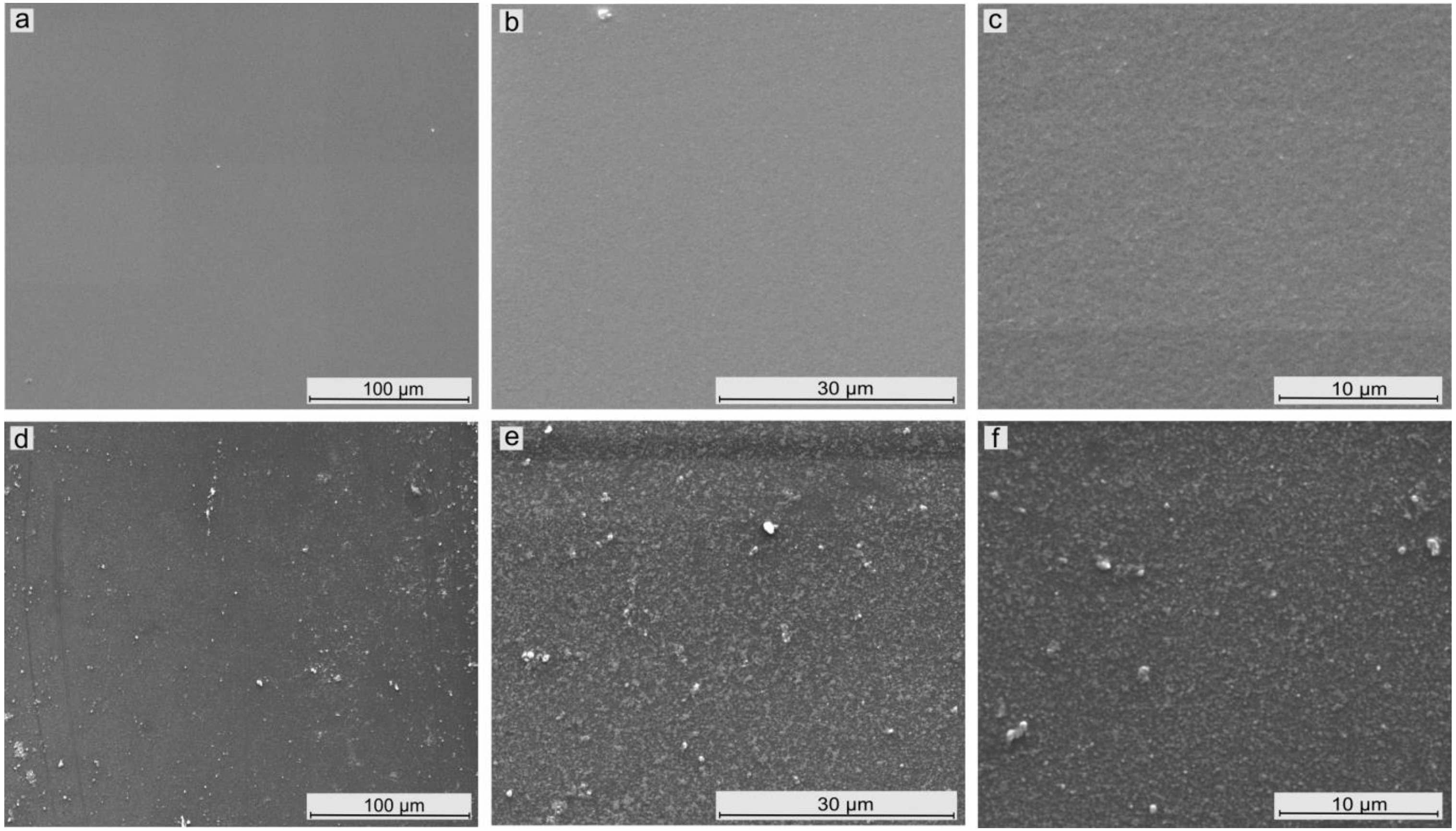

Fig. SI2. SEM images of (a, b, c) ZnO layer on ITO-coated glass and (d, e, f) P3HT:CNO **4** layer spin-coated on the ZnO/ITO with different magnification: (a, d) 1000x, (b, e) 5000x and (c, f) 10000x. For better quality of SEM images, Au (3 nm thickness layer) was sputtered on the surface.

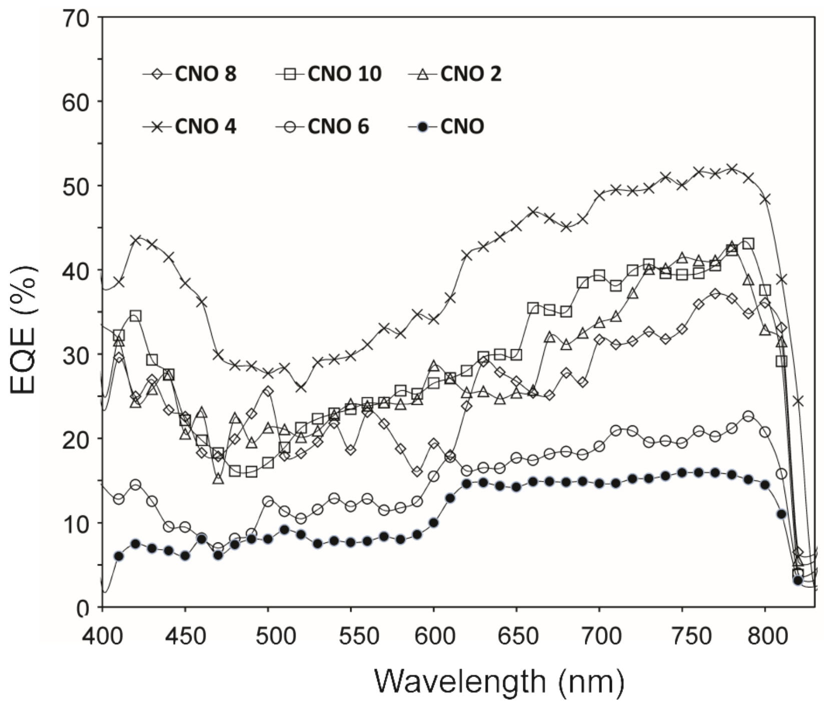

Fig. SI3. Photoconversion efficiency study - external quantum efficiency for devices based on the CNO derivatives (CNO **2**, CNO **4**, CNO **6**, CNO **8** and CNO **10**).
